# ESPO/BEHAVIORAL AND SOCIAL SCIENCES: SOCIAL RELATIONSHIPS, ISOLATION, AND WELL-BEING

**DOI:** 10.1093/geroni/igz038.224

**Published:** 2019-11-08

**Authors:** Jeffrey E Stokes, Elliane Irani, Patricia A Thomas

**Affiliations:** 1 University of Massachusetts Boston, Boston, Massachusetts, United States; 2 Case Western Reserve University, Frances Payne Bolton School of Nursing, Cleveland, Ohio, United States; 3 Case Western Reserve University School of Medicine, Cleveland, Ohio, United States

## Abstract

The purpose of this symposium is twofold: (1) To present innovative research linking social relationships, isolation, and well-being among older adults, and (2) To highlight new and emerging scholars in the Behavioral and Social Sciences section of GSA. The papers in this symposium examine the repercussions of numerous relationships for well-being in later life. Huo and colleagues examine the impacts of contact with close and not-close social partners on physical activity, highlighting differences by gender. Polenick and colleagues focus on perhaps the closest of relationships in later life: marriage, analyzing longitudinal associations between discordant chronic conditions and depressive symptoms among older couples. Upenieks takes an intergenerational perspective, examining the embeddedness of adult children in older adults’ networks in the context of both depression onset and chronically high depressive symptomology. This paper also highlights the consequences of well-being for older adults’ social isolation, and not merely the reverse. Hladek and colleagues explore the subjective side of isolation among older adults with chronic disease, noting links between loneliness and self-efficacy that may have clinical and interventional significance. Lastly, Meinertz and Gilligan explore potential gaps in service provision that may increase rural older adults’ risk of isolation and abuse. Taken together, these five papers underscore the importance of various social relationships for older adults’ well-being, and suggest implications for how best to promote healthy aging. As discussant, Thomas will assess the strengths and limitations of these papers, and consider the contributions these studies – and new scholars – can make to the field.

